# Inflammatory and redox responses associated with sequential exposure to poultry-house PM2.5-derived material and avian pathogenic *Escherichia coli* in chicken models

**DOI:** 10.1016/j.psj.2026.108064

**Published:** 2026-10-31

**Authors:** Luna Li, Cheng Bao, Chong Liu, Linlin Jiang, Jiao Wang, Xin Yu, Xinye Wang, Dechen Lu, Guozhong Chen, Hongwei Zhu, Haoxiang Li, Ziru Sun, Xingxiao Zhang, Jianlong Zhang

**Affiliations:** a School of Life Sciences, Ludong University, Yantai, 264025, China; b Collaborative Innovation Center for the Pet Infectious Diseases and Public Health in the Middle and Lower Stream Regions of the Yellow River, Yantai, 264025, China; c Shandong Engineering Research Center for Aquaculture Environment Control, Yantai, 264025, China; d Yantai Key Laboratory of Animal Pathogenic Microbiology and Immunology, Yantai, 264025, China

**Keywords:** Avian pathogenic *Escherichia coli*, Chicken, Cystic fibrosis transmembrane conductance regulator, Particulate matter, Respiratory inflammation

## Abstract

Fine particulate matter collected in poultry houses and avian pathogenic *Escherichia coli* (APEC) are respiratory stressors in chickens, but responses to an ordered exposure schedule are incompletely defined. We examined material collected in the PM2.5 fraction of a broiler house and subsequently eluted, lyophilized, and autoclaved (PM2.5-derived material) together with an APEC O1:K1 isolate. Chicken embryonic type II-like lung epithelial cells were assigned to control, processed PM2.5-derived material, APEC, or PM2.5-followed-by-APEC schedules; N-acetyl-L-cysteine (NAC) and 4-chlorobenzo[f]isoquinoline (CBIQ) were used as pharmacological interventions. In vivo, 40 supplier-certified specific-pathogen-free chicks (arrived at 7 d of age; treatment began at 10 d of age; n = 10/group) received control, single-exposure, or sequential schedules for 14 d. Under these prespecified schedules, the sequential group showed the largest changes for several inflammatory and redox-related endpoints, lower cystic fibrosis transmembrane conductance regulator (CFTR) abundance, altered total abundance of PI3K, AKT, mTOR, IκBα, and NF-κB p65, and greater accumulation of LC3B- and MDC-associated markers. NAC and CBIQ shifted selected cellular responses toward control values. Serum inflammatory/redox indices and qualitative lung histology in chicks were directionally consistent with selected cellular findings. The processed material was not characterized after sterilization for particle-size distribution, elemental/ionic composition, or endotoxin content, and the exposure duration and timing differed among schedules. Therefore, these data describe associations under the tested schedules and do not establish a PM2.5 × APEC interaction, statistical synergy, causal priming, particle-specific effects, or pathway activation. Future factorial studies using fully characterized particulate preparations and clinical and microbiological endpoints are required.

## Introduction

Particulate matter in intensive poultry-production facilities originates from feed, litter, feather debris, dander, excreta, and associated microbial material. Fine particles with an aerodynamic diameter of 2.5 μm or less (PM2.5) can penetrate the respiratory tract and have been associated with oxidative stress, epithelial-barrier disruption, and dysregulated immune responses (Aghapour et al., 2022; Wang et al., 2023). Poultry-house PM2.5 is a complex exposure rather than an inert particle fraction: broiler-house PM2.5 contains organic and inorganic constituents, microorganisms, and measurable endotoxin (Dai et al., 2020), and recent Poultry Science studies further document diverse bacterial and fungal communities within poultry-house PM2.5 (Wang et al., 2024). In broilers, inhaled or house-derived PM2.5 has been associated with lung injury and altered pulmonary or gut microbial communities (Shen et al., 2023; Zhou et al., 2023, 2024). These observations support evaluating poultry-house PM2.5 as a mixed environmental exposure that may modify respiratory responses to subsequent infectious challenge.

Avian pathogenic *Escherichia coli* (APEC), a major cause of avian colibacillosis, can produce airsacculitis, pneumonia, pericarditis, perihepatitis, and septicemia. Disease expression depends on bacterial virulence, host status, age, and environmental or infectious stressors (Dho-Moulin and Fairbrother, 1999; Alber et al., 2021; Nawaz et al., 2024). Beyond serving as a physical barrier, the pulmonary epithelium senses inhaled particles and microorganisms and releases cytokines and chemokines. Chicken lung epithelial cell models are established tools for respiratory-infection research (Esnault et al., 2011; Meyer et al., 2017), and APEC can adhere to and invade chicken lung epithelial cells and induce host-response programs (Mol et al., 2019).

Cystic fibrosis transmembrane conductance regulator (CFTR) is a cAMP-regulated anion channel that contributes to epithelial ion transport, airway surface liquid homeostasis, and inflammatory regulation (Saint-Criq and Gray, 2017). Exposure to air pollutants can reduce CFTR expression or function in respiratory epithelia and is associated with oxidative stress and altered inflammatory signaling (Nguyen et al., 2021; Stermann et al., 2022; Liu et al., 2024). PI3K/AKT/mTOR and NF-κB signaling, together with autophagy-related processes, have been implicated in particle-induced injury in chicken and mammalian epithelial models (Su et al., 2017; Zhu et al., 2018; Han and Zhuang, 2021; Xi et al., 2022).

Previous studies have reported enhanced inflammatory responses when poultry-house PM2.5 is combined with *Pseudomonas aeruginosa* in experimental systems (Li et al., 2020; Zhang et al., 2022). Whether a PM2.5-first schedule is associated with a distinct response to subsequent APEC exposure in chicken pulmonary epithelium remains incompletely defined. Accordingly, we compared prespecified control, PM2.5-only, APEC-only, and PM2.5-followed-by-APEC schedules in chicken embryonic type II-like lung epithelial cells and chicks. We also used NAC and CBIQ to test whether selected redox- and CFTR-related responses were pharmacologically modifiable. We hypothesized that the sequential schedule would be associated with a more pronounced pattern for selected inflammatory and redox-related endpoints and with coordinated changes in CFTR expression, PI3K/AKT/mTOR-related total-protein abundance, NF-κB-related expression, and autophagy-associated markers. This was a schedule-comparison study rather than a matched factorial experiment; therefore, the design was not intended to estimate a PM2.5 × APEC interaction, statistical synergy, or causal priming.

## Materials and methods

### Ethics approval, study design, and chicks

All animal procedures were reviewed and approved by the Animal Ethics and Experimentation Committee of Ludong University (approval no. LDU-IACUC2020001) and were conducted in accordance with institutional guidelines for the care and use of experimental animals. The reported PM2.5 sampling campaigns were environmental collections performed in 2022; the animal procedures and embryonic-cell isolation used in this study were conducted under the approved protocol.

Forty supplier-certified specific-pathogen-free (SPF) Yisheng 909 commercial-line chicks, with equal numbers of males and females, were obtained from Shandong Yisheng Livestock and Poultry Breeding Co., Ltd. (Yantai, China). Chicks arrived at 7 d of age and, after a 3-d acclimation, were assigned by a random-number table to four treatment groups (n = 10 per group): control, PM2.5, APEC, and PM2.5 followed by APEC (sequential exposure). Treatment began at 10 d of age; thus, d 1 of the exposure period corresponds to 10 d of age. Chicks were housed in SPF isolators under barrier conditions (brooding temperature 32 to 33°C, reduced by 3°C each week; relative humidity 55 to 65%; lighting schedule 23L:1D) with ad libitum access to sterilized complete SPF feed and sterilized tap water. “SPF” refers only to the supplier’s certified pathogen-exclusion status and should not be interpreted as culture- or PCR-confirmed absence of commensal or potentially pathogenic *Escherichia coli*; baseline *Escherichia coli*/APEC carriage was not tested in this study.

The treatment schedules were: control, phosphate-buffered saline (PBS; 200 μL/chick) on d 1 to 14; PM2.5 group, processed PM2.5-derived material at 1 mg/mL (200 μL/chick, equivalent to 0.20 mg per administration) on d 1 to 14; APEC group, 1 × 10⁸ CFU/mL bacterial suspension (200 μL/chick, equivalent to 2 × 10⁷ CFU per administration) on d 1 to 14; and sequential-exposure group, processed PM2.5-derived material at the same concentration and volume on d 1 to 7 followed by APEC at the same concentration and volume on d 8 to 14. Administration was performed by slow dropwise instillation at the nares, 100 μL per nostril (200 μL per chick), with each chick held upright and allowed to inhale the drops; comparable 100-μL-per-nostril intranasal delivery has been described in chickens (Kang et al., 2013). No tracheal cannulation was used. Clinical status was checked daily as part of routine monitoring. At the end of the 14-d exposure period, chicks were euthanized according to the approved protocol, and blood and lung tissues were collected. Because the single-exposure groups received an agent on d 1 to 14 whereas the sequential group received each agent for 7 d, exposure duration and timing were not matched across schedules.

### Collection and preparation of poultry-house PM2.5

PM2.5 was collected from a broiler house in Fushan District, Yantai, Shandong Province, China (37°24′22.91″ N, 121°21′36.30″ E) during two independent campaigns (June 24 to July 24, 2022, and August 24 to September 24, 2022). During each campaign, an airborne-particle sampler (ZR-3920, Qingdao Junray Intelligent Instrument Co., Ltd., Qingdao, China) was positioned at the center of the house 1 m above the floor and operated at 100 L/min. Material in the PM2.5 sampling fraction was collected on 9 × 9 cm waterproof glass-fiber filters that had been dry-heat treated before sampling. Each filter was used continuously for approximately 72 h and then replaced until the end of the campaign. After each campaign, filters were pooled and ultrasonically eluted in sterile double-distilled water three times for 15 min each. The pooled eluate was frozen overnight at −80°C and lyophilized for 24 h. Equal masses from the two campaigns were combined and suspended in sterile, pyrogen-free PBS at 10 mg/mL; stock suspensions were stored short-term at 4°C, vortexed before use, and autoclaved before administration. Endotoxin was not quantified, and autoclaving was used for sterilization rather than depyrogenation; heat-stable lipopolysaccharide could therefore remain. Particle-size distribution, elemental/ionic composition, and microbiological/endotoxin content were not characterized after processing. Accordingly, “processed PM2.5-derived material” denotes material recovered from the original PM2.5 sampling fraction and does not assert that the post-processing aerodynamic size distribution remained ≤2.5 μm. The abbreviated group label “PM2.5″ is retained in figures and tables for readability.

### APEC culture

An avian pathogenic *Escherichia coli* O1:K1 strain maintained in our laboratory was used. The strain was originally isolated from the lung of a chicken with colibacillosis-associated septicemia and was identified as APEC by serotyping and whole-genome sequencing. Comparative genomic analysis showed high consistency with the reference genome of *Escherichia coli* APEC O1 in the KEGG Genome database. After revival, bacteria were streaked on Luria-Bertani (LB) agar and incubated in an inverted position at 37°C for 12 h. A single colony was transferred to LB broth and cultured at 37°C with shaking at 180 rpm for 8 h to reach logarithmic growth. Bacterial concentration was estimated from optical density at 600 nm (OD600) using a laboratory-established standard curve (OD600 = 1.0, approximately 1.0 × 10⁹ CFU/mL). Before each experiment, diluted inocula were plated on LB agar for viable counting; the actual inoculum was required to be within ±0.5 log10 CFU/mL of the target. Bacteria were diluted in sterile, pyrogen-free PBS to 1 × 10⁸ CFU/mL and used immediately.

### Isolation and culture of chicken embryonic type II-like lung epithelial cells

Lungs were aseptically collected from ten 18-d-old SPF chicken embryos after euthanasia according to the approved protocol. Nonpulmonary tissues were removed, and lung tissue was minced and washed with PBS until the wash solution was clear. Tissue was digested with 0.25% trypsin at 37°C in 5% CO₂ for 10 min with gentle mixing every 2 min. Digestion was terminated by adding an equal volume of Dulbecco’s modified Eagle medium (DMEM) containing 10% fetal bovine serum (FBS), followed by centrifugation at 800 rpm for 10 min. The pellet was further digested with 0.1% type IV collagenase for 20 min, passed sequentially through 100- and 70-μm cell strainers, and centrifuged at 1,000 rpm for 5 min under refrigerated conditions.

Cells were resuspended in DMEM containing 10% FBS, and fibroblast contamination was reduced by three rounds of differential adherence (45 min each). Nonadherent cells were transferred to chicken IgG-coated culture dishes (1 mg/mL; coated for 2.5 h at 37°C) and incubated for 1 h to reduce macrophage-like cell contamination. Unbound cells were passed through a 40-μm strainer, seeded at 1 × 10⁶ cells/mL, and cultured at 37°C in 5% CO₂. The medium was replaced with DMEM containing 10% FBS after 15 to 18 h. The phenotype of the isolated cells was initially evaluated using alkaline phosphatase BCIP/NBT staining. To further characterize the cell population, the expression of four alveolar type II epithelial cell-associated marker genes was examined using reverse transcription PCR (RT-PCR) followed by agarose gel electrophoresis.

Three independent cell-isolation experiments were performed on different dates using separate batches of SPF chicken embryos. Cells from each isolation were processed and analyzed independently. For assays performed in plate format, each treatment included 3 technical replicate wells within each independent experiment, and technical replicates were averaged before statistical analysis. Each independent cell-isolation experiment was considered one biological replicate (n = 3), and statistical analyses were performed using values derived from independent isolation batches rather than individual technical wells.

### RT-PCR and agarose gel electrophoresis for cell phenotypic characterization

Total RNA was extracted from the isolated chicken embryonic lung epithelial cell preparations according to the manufacturer's instructions. Equal amounts of total RNA were reverse-transcribed into complementary DNA (cDNA) using a commercial reverse-transcription kit. Four alveolar type II epithelial cell-associated marker genes, including lysosomal associated membrane protein 3 (LAMP3), surfactant protein C (SFTPC), surfactant protein A1 (SFTPA1), and fatty acid binding protein 3 (FABP3), were examined using reverse transcription PCR (RT-PCR) followed by agarose gel electrophoresis. Primer sequences are listed in Table 1.

**Table 1:** Primers used for quantitative real-time PCR and endpoint RT-PCR.

**Gene**	**Forward primer (5′–3′)**	**Reverse primer (5′–3′)**	**Product size (bp)**	**Accession no.**
CFTR	GCAATTTTCCCCTTCATGCTT	AAGTCTTGCTGATCAGT	195	NM_001105666.2
PI3K	GCTAATAAACCACTACCGGAA	TAATTTCTTACCCACCGCTTC	153	NM_001004410.2
AKT	TATTCTTTTCAGACACACAGA	CTTCAGAAAATACACGCTCT	90	NM_205055.1
mTOR	TTCCTCACATGTTACGGGTC	TAGTCATCCAGGTTAGCTCC	127	XM_417614.6
NF-κB p65	CAGCCCATCTATGACAACCG	TCAGCCCAGAAACGAACCTC	90	NM_001396038.1
LAMP3	GCAGTCCCCAGGAAACCTTTA	GTATGGTGGAAGGACGTGGTT	200	NM_001146132.2
SFTPC	GCACCACCATCAACATCCTCT	GGCACTCAGACACAGCCTTTA	164	XM_025142875.1
SFTPA1	TTTCCCATCAGTTGCCGGTAG	TCCTGGCACATTTTCACCGTA	177	NM_001039166.1
FABP3	GGTGACGGTGAAGACCCATAG	CACTAGCGATGTCTCCTTCCC	172	NM_001030889.2
GAPDH	GTCCTCTCTGGGAAAGTCC	TGATCACAAGTTTCCCGTTC	102	NM_204305.2

Note: All primers were designed based on Gallus gallus sequences retrieved from NCBI using Primer-BLAST, and their sequence specificity was assessed by BLAST. For quantitative real-time PCR primers, amplification efficiency (90–110%) and single-product specificity were evaluated using standard-curve and melting-curve analyses, respectively. For endpoint RT-PCR primers targeting LAMP3, SFTPC, SFTPA1, and FABP3, the expected amplification products were assessed by agarose gel electrophoresis.

Each PCR reaction was performed in a total volume of 20 μL containing 10.0 μL of 2 × Taq PCR Master Mix, 0.4 μL of forward primer (10 μmol/L), 0.4 μL of reverse primer (10 μmol/L), 1.0 μL of cDNA template, and 8.2 μL of nuclease-free water. The amplification conditions consisted of an initial denaturation at 95°C for 3 min, followed by 35 cycles of denaturation at 95°C for 15 s, annealing at 60°C for 20 s, and extension at 72°C for 20 s, with a final extension at 72°C for 5 min.

The amplified products were separated by electrophoresis on a 2% agarose gel using a 100-bp DNA ladder as the molecular size marker. DNA bands were visualized and recorded using a gel documentation system. The presence of amplification products at the expected sizes was used as supplementary transcript-level evidence for the type II-associated phenotype of the isolated cells.

### Cell exposure and pharmacological interventions

Cells were assigned to control, PM2.5, APEC, or sequential PM2.5-followed-by-APEC schedules. Processed PM2.5-derived material was used at 0.50 mg/mL, and APEC was used at a multiplicity of infection (MOI) of 100:1. Control cultures received the corresponding vehicle/medium and were harvested at 4.5 h. PM2.5-only and APEC-only cultures were exposed to their respective agent over the 4.5-h experimental interval. For sequential exposure, cells received processed PM2.5-derived material for 2 h and were then exposed to APEC for an additional 2.5 h; samples were collected at 4.5 h. Thus, although the harvest time was common, agent-specific exposure duration was not matched among the single and sequential schedules. The PM2.5 concentration and 2-h + 2.5-h sequence were selected from published lung-epithelial PM exposure studies and preliminary dose-response optimization in our system that yielded detectable, non-saturating inflammatory and redox-related responses; these preliminary observations were not a formal cell-viability assessment. The design compares prespecified schedules and does not estimate a matched-dose PM2.5 × APEC interaction.

To test whether selected sequential-exposure-associated responses were pharmacologically modifiable, cells were pretreated with N-acetyl-L-cysteine (NAC) or 4-chlorobenzo[f]isoquinoline (CBIQ). NAC was used as a thiol-containing antioxidant. CBIQ was used as a CFTR activator/modulator, although it is not completely selective for CFTR (Murthy et al., 2005). NAC (YT305, Beijing BioLab Technology Co., Ltd., Beijing, China) was prepared as a 100 mM stock in sterile PBS, diluted in DMEM to 5 mM, and added 1 h before PM2.5 exposure; corresponding controls received an equivalent volume of PBS.

CBIQ (C6617, Sigma-Aldrich, St. Louis, MO) was prepared as a 10 mM stock in dimethyl sulfoxide (DMSO) and diluted in DMEM to 10 μM. CBIQ was added 1 h before PM2.5 exposure, and corresponding controls received an equivalent volume of DMSO (<0.1%). Vehicle controls were included in all intervention experiments.

Each treatment was performed in 3 technical replicate wells within each independent cell-isolation experiment, and the experiments were independently repeated 3 times using separate cell preparations. Technical replicates were averaged before statistical analysis, and each independent cell-isolation experiment was considered one biological replicate.

### Inflammatory, oxidative stress, and cell injury-related measurements

Cells were seeded in 6-well plates at a density of 1 × 10⁶ cells/well, and culture supernatants were collected after the indicated treatments. The concentrations of inflammatory mediators, including tumor necrosis factor-α (TNF-α; CB10160-Ch), interleukin-1β (IL-1β; CB10037-Ch), and monocyte chemoattractant protein-1 (MCP-1; CB10411-Ch), were measured using commercial ELISA kits (Shanghai Coibo Biotechnology Co., Ltd., Shanghai, China) according to the manufacturer’s instructions.

Oxidative/redox-related measurements included reactive oxygen species (ROS; CB10086-Ch), superoxide dismutase (SOD; CB10054-Ch), catalase (CAT; CB10279-Ch), and glutathione (GSH; CB10280-Ch); aspartate aminotransferase (AST; CB10186-Ch) was measured as an injury-associated biochemical indicator. These kits were obtained from Shanghai Coibo Biotechnology Co., Ltd. In chick serum, TNF-α, IL-1β, MCP-1, and ROS-associated signals were measured using the corresponding commercial assay kits according to the manufacturer's instructions. Total antioxidant capacity (T-AOC) was determined exclusively in serum using a commercial chicken T-AOC ELISA kit (catalog no. CB10167-Ch; Shanghai Coibo Biotechnology Co., Ltd., Shanghai, China). According to the manufacturer's assay description, the kit employs an antibody-based enzyme-linked immunosorbent assay involving biotinylated detection antibodies, horseradish peroxidase (HRP)-mediated color development, and 3,3′,5,5′-tetramethylbenzidine (TMB) as the chromogenic substrate. Absorbance was measured at 450 nm, and sample values were calculated from a standard calibration curve constructed using the concentrations and corresponding optical density values of the provided standards. Serum T-AOC results were expressed in U/mL. T-AOC was not measured in cell culture supernatants.

All assays were performed according to the manufacturers' instructions. Sample dilution factors and detection ranges followed the corresponding kit specifications. TNF-α, IL-1β, and MCP-1 concentrations were expressed as pg/mL, while serum T-AOC values were reported in U/mL. Other biochemical indicators were expressed in the units specified by their respective assay kits.

### Quantitative real-time PCR

Total RNA was extracted from cell samples using TRIzol reagent (Sigma-Aldrich, St. Louis, MO) according to the manufacturer’s instructions. RNA concentration and purity were determined spectrophotometrically. Samples with OD260/280 ratios of 1.8 to 2.0 and OD260/230 ratios of 2.0 to 2.2 were used for reverse transcription. Complementary DNA was synthesized using an M-MLV reverse-transcription kit (Thermo Fisher Scientific, Waltham, MA). Quantitative real-time PCR was performed with SYBR Green reagents from Vazyme (Nanjing, China).

The amplification program consisted of an initial denaturation at 95°C for 30 s, followed by 40 cycles of amplification at 95°C for 10 s and 60°C for 30 s. A melting-curve analysis was performed to confirm amplification specificity. Relative transcript abundance was calculated using the 2^−ΔΔCt method and normalized to GAPDH expression. Primer sequences are listed in Table 1. All primers were newly designed for this study based on *Gallus gallus* sequences (NCBI) using Primer-BLAST; specificity was confirmed by BLAST, and amplification efficiency (90–110%) and single-product specificity were validated by standard and melting-curve analyses.

Each sample was analyzed using technical replicates, and the mean Ct value was used for subsequent calculations. The stability of GAPDH expression among experimental groups was confirmed before relative quantification.

### Western blot analysis

Cells were lysed on ice for 30 min in radioimmunoprecipitation assay (RIPA) buffer containing protease inhibitors and phenylmethylsulfonyl fluoride, followed by centrifugation at 12,000 rpm for 15 min at 4°C. Protein concentration was determined by bicinchoninic acid assay. Equal amounts of protein were separated by sodium dodecyl sulfate-polyacrylamide gel electrophoresis (SDS-PAGE) and transferred to polyvinylidene difluoride (PVDF) membranes.

Membranes were blocked for 2 h at room temperature with Tris-buffered saline containing Tween 20 (TBST) and 5% nonfat dry milk and incubated overnight at 4°C with primary antibodies against CFTR (20738-1-AP, rabbit, 1:1,000, 168 kDa; Proteintech, Rosemont, IL), PI3K (4257, rabbit, 1:1,000, 85 kDa; Cell Signaling Technology, Danvers, MA), AKT (4691, rabbit, 1:1,000, 60 kDa; Cell Signaling Technology), mTOR (2983, rabbit, 1:1,000, 289 kDa; Cell Signaling Technology), LC3B (2775, rabbit, 1:1,000, 14/16 kDa; Cell Signaling Technology), IκBα (4814, rabbit, 1:1,000, 39 kDa; Cell Signaling Technology), NF-κB p65 (8242, rabbit, 1:1,000, 65 kDa; Cell Signaling Technology), and β-actin (66009-1-Ig, mouse, 1:5,000, 42 kDa; Proteintech).

After washing with TBST, membranes were incubated for 1 h at room temperature with horseradish peroxidase-conjugated goat anti-rabbit IgG (7074, 1:2,000; Cell Signaling Technology) or goat anti-mouse IgG (7076, 1:2,000; Cell Signaling Technology). Immunoreactive bands were visualized using enhanced chemiluminescence and captured using a gel documentation system. Band intensities were quantified using ImageJ software and normalized to β-actin expression.

Densitometric analysis used 20 μg protein per lane and exposures within the nonsaturated detection range. Band intensity was normalized to β-actin. Each independent cell-isolation experiment was treated as one biological replicate, and technical replicates were averaged before analysis.

### Monodansylcadaverine staining

After treatment, cells were fixed in 1% paraformaldehyde for 15 min at 37°C and stained using a monodansylcadaverine (MDC) autophagy staining kit (Beyotime Biotechnology, Shanghai, China) according to the manufacturer’s instructions. Fluorescence signals were observed and recorded using a fluorescence microscope. MDC staining and LC3B abundance were evaluated as autophagy-associated readouts.

### Lung histopathology

Lung tissues were fixed in 4% paraformaldehyde for 48 h, dehydrated through graded ethanol (70, 80, 95, and 100%), cleared in xylene, embedded in paraffin, and sectioned at 4 μm. Sections were deparaffinized, rehydrated, stained with hematoxylin and eosin (H&E), and examined by light microscopy. Images were acquired at 100 × magnification; the corresponding scale bars used for these lung images were 200 μm. Histology was evaluated descriptively. A prospective per-bird histologic lesion-scoring scheme and standardized gross/airsac lesion score were not part of the original protocol; therefore, no retrospective numerical lesion score is reported from representative archived fields.

### Statistical analysis

Data were analyzed using GraphPad Prism 7.04 and are presented as mean ± SD. Comparisons among groups were performed using one-way analysis of variance (ANOVA) followed by Tukey’s multiple comparison test. Differences were considered statistically significant at P < 0.05. For cell experiments, each independent cell-isolation batch was considered one biological replicate, and technical replicate wells were averaged before statistical analysis. For chick experiments, each individual chick was considered the experimental unit.

## Results

### Inflammatory and redox-related responses under the tested PM2.5 and APEC exposure schedules

Exposure to either PM2.5-derived material or APEC alone was associated with higher TNF-α, IL-1β, and MCP-1 concentrations in cell-culture supernatants than the control treatment (Figure 1A–C). Under the sequential schedule, TNF-α was higher than in the control and both single-exposure groups (Figure 1A). IL-1β was higher than in the control, and the significant between-group comparisons shown in Figure 1B also included the sequential and corresponding single-exposure groups. MCP-1 was higher in the sequential group than in the control and PM2.5-only groups (Figure 1C). These comparisons describe the tested schedules and are not estimates of a factorial PM2.5 × APEC interaction.

**Figure 1:**
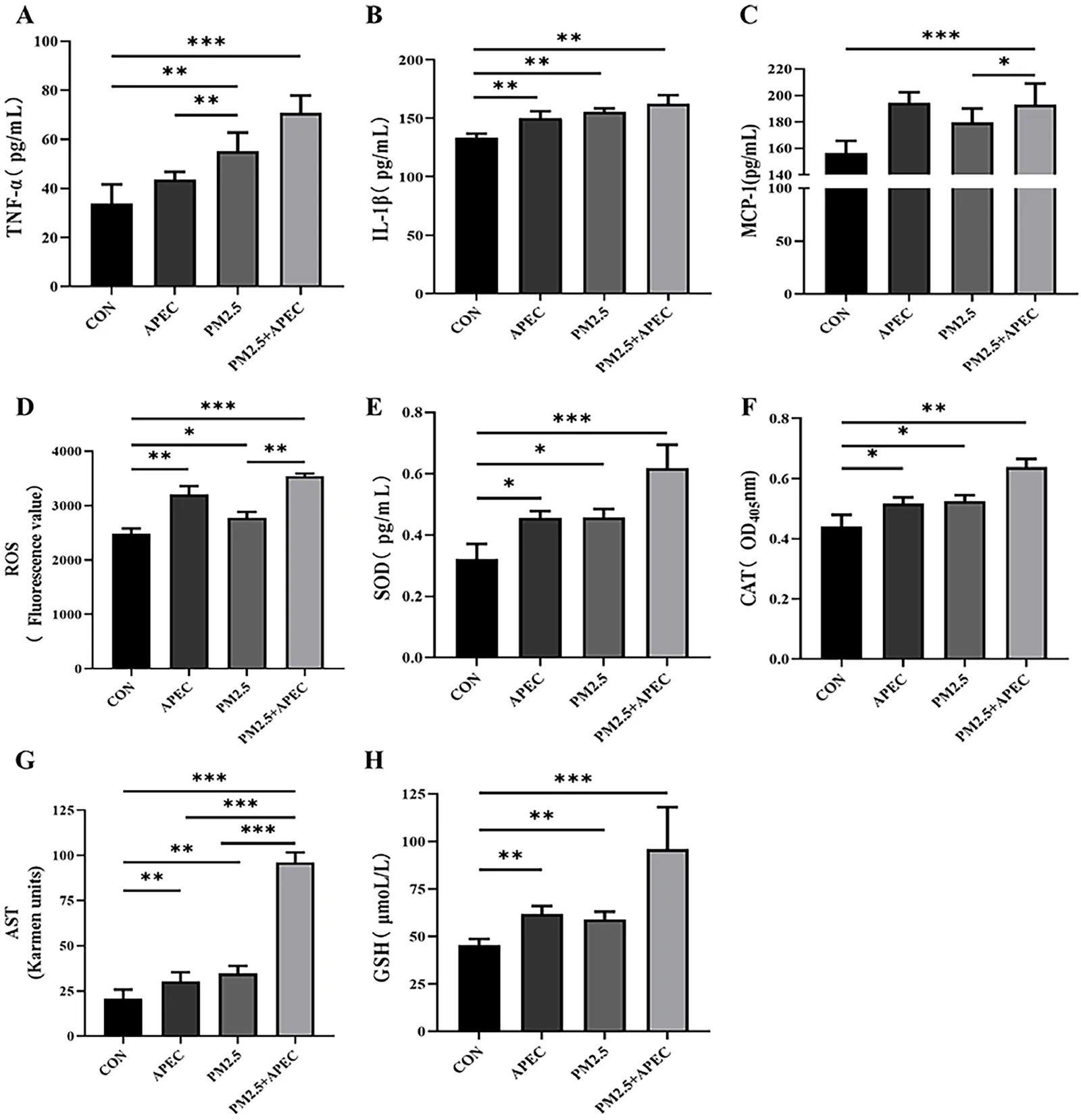
Inflammatory, redox-related, and injury-associated responses under the tested PM2.5-derived material and APEC exposure schedules in chicken embryonic type II-like lung epithelial cells. (A–C) Concentrations of TNF-α, IL-1β, and MCP-1 in cell-culture supernatants. (D) ROS-associated fluorescence signal. (E–H) Activities or levels of SOD, CAT, AST, and GSH, respectively. Cells were assigned to the control, PM2.5, APEC, or sequential PM2.5-followed-by-APEC groups. Data are presented as mean ± SD from 3 independent cell-isolation experiments. Technical replicate wells within each independent isolation were averaged before statistical analysis, and each independent cell-isolation experiment was considered one biological replicate (n = 3). Statistical comparisons were performed using one-way ANOVA followed by Tukey’s multiple comparison test. Brackets indicate the pairwise comparisons shown in the figure. *P < 0.05, **P < 0.01, and ***P < 0.001. PM2.5, particulate matter with an aerodynamic diameter ≤2.5 μm at collection; APEC, avian pathogenic *Escherichia coli*; AST, aspartate aminotransferase; CAT, catalase; GSH, glutathione; MCP-1, monocyte chemoattractant protein 1; ROS, reactive oxygen species-associated fluorescence signal; SOD, superoxide dismutase; TNF-α, tumor necrosis factor α.

All exposure schedules were associated with a higher ROS-associated signal than the control, and the highest mean signal occurred in the sequential group, with significant differences from the control and single-exposure groups (Figure 1D). SOD and CAT activities were higher in exposed cells, with the highest mean values in the sequential group (Figure 1E and F). GSH levels were also higher under the sequential schedule (Figure 1H). AST, used here as an injury-associated biochemical indicator rather than a viability measurement, was higher after sequential exposure than in the control or either single-exposure group (Figure 1G).

### Sequential PM2.5 followed by APEC is associated with altered CFTR- and inflammation-related transcript and total-protein abundance

Under the sequential schedule, CFTR transcript and total-protein abundance were lower than in the control group (Figure 2A, F, and G). PM2.5-derived material or APEC alone also decreased CFTR abundance relative to the control, although the magnitude differed between schedules. The lowest mean CFTR abundance among exposed groups occurred in the sequential group.

**Figure 2:**
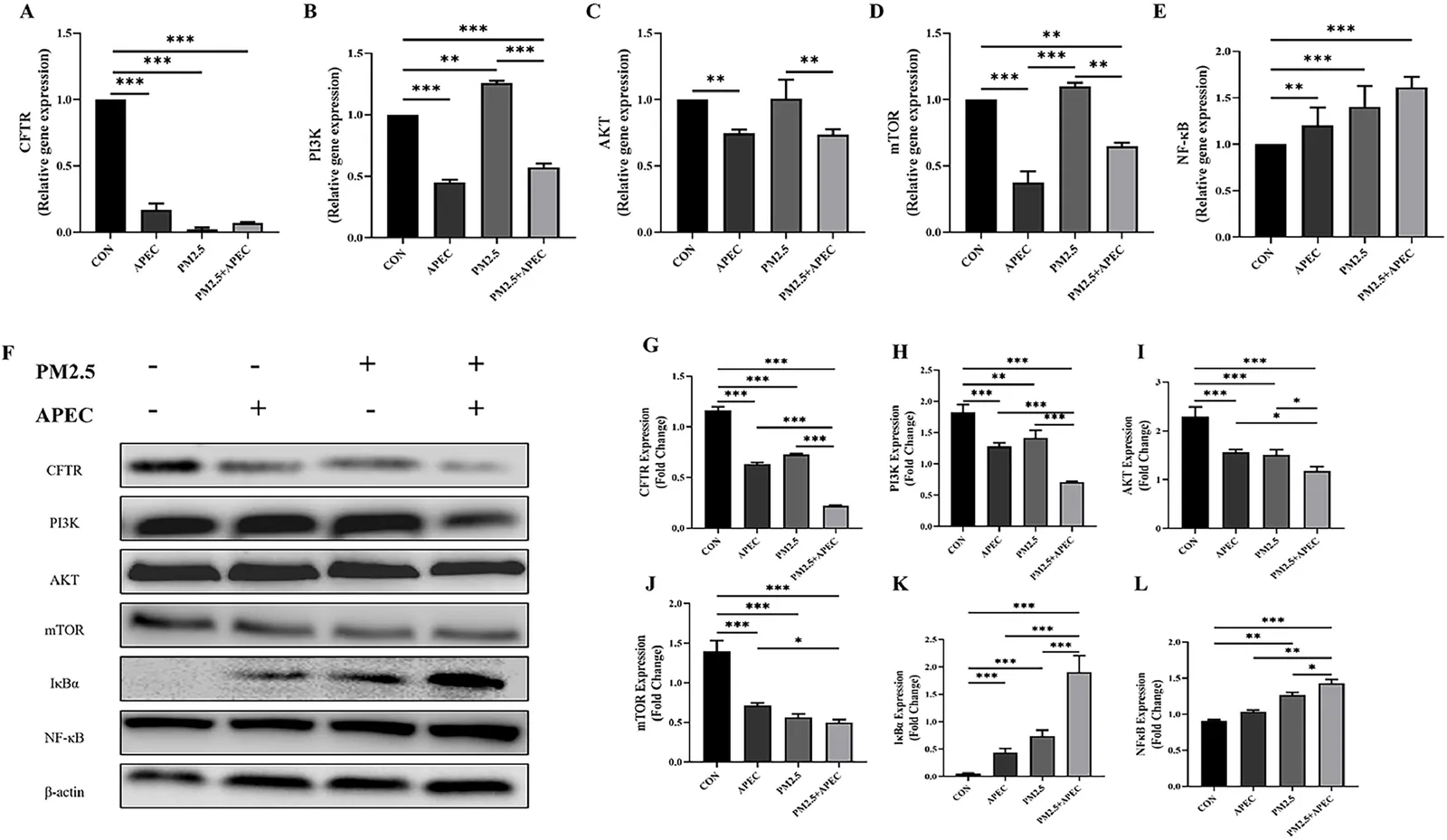
CFTR-related and inflammation-related transcript and total-protein abundance under the tested PM2.5-derived material and APEC exposure schedules in chicken embryonic type II-like lung epithelial cells. (A–E) Relative transcript abundance of CFTR, PI3K, AKT, mTOR, and NF-κB p65, respectively. (F) Representative Western blot images of CFTR, PI3K, AKT, mTOR, IκBα, NF-κB p65, and β-actin. (G–L) Densitometric quantification of CFTR, PI3K, AKT, mTOR, IκBα, and NF-κB p65 protein abundance, respectively, normalized to β-actin. PI3K, AKT, and mTOR measurements represent total protein abundance and do not directly reflect phosphorylation status or pathway activity. Data are presented as mean ± SD from 3 independent cell-isolation experiments. Technical replicate wells within each independent isolation were averaged before statistical analysis, and each independent isolation was considered one biological replicate (n = 3). Statistical comparisons were performed using one-way ANOVA followed by Tukey’s multiple comparison test. *P < 0.05, **P < 0.01, and ***P < 0.001.

PI3K, AKT, and mTOR transcript abundance and total-protein abundance were lower than in the control across the exposure schedules (Figure 2B–D, F, and H–J). The sequential group showed lower abundance of these molecules than the control, with additional significant differences from selected single-exposure groups as indicated in the corresponding figure panels.

NF-κB p65 transcript abundance and total NF-κB p65 protein abundance were higher after sequential exposure than in the control group (Figure 2E, F, and L). Total IκBα protein abundance was also higher in the sequential group than in the control (Figure 2F and K). These measurements demonstrate differences in abundance; without phosphorylation or nuclear-translocation measurements, they do not demonstrate NF-κB activation.

Together, the data show that the prespecified PM2.5-followed-by-APEC schedule was associated with changes in CFTR, PI3K, AKT, mTOR, IκBα, and NF-κB p65 transcript and/or total-protein abundance. The measurements describe molecular-expression profiles and do not directly measure pathway activity.

### Sequential PM2.5 followed by APEC is associated with accumulation of autophagy-related markers

LC3B protein abundance differed among the exposure groups, with the highest signal observed after sequential PM2.5-followed-by-APEC exposure (Figure 3A and B). LC3B abundance in the sequential-exposure group was greater than that in the control and both single-exposure groups.

**Figure 3:**
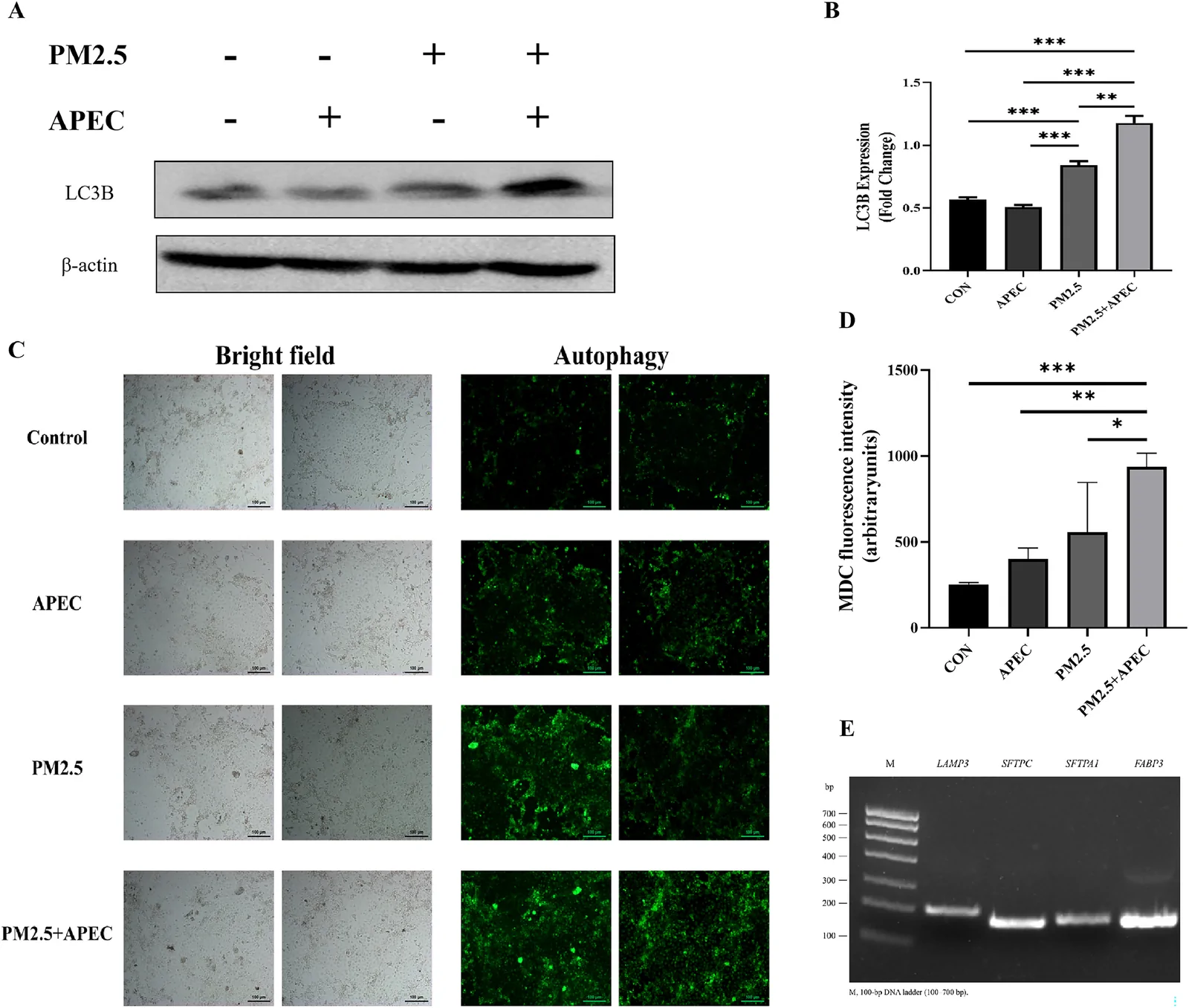
Autophagy-associated readouts and phenotypic marker expression in chicken embryonic type II-like lung epithelial cells. (A) Representative Western blot images of LC3B and β-actin after control, PM2.5, APEC, or sequential PM2.5-followed-by-APEC treatment. (B) Densitometric quantification of LC3B total-protein abundance normalized to β-actin. (C) Representative bright-field and monodansylcadaverine (MDC) fluorescence images of cells under the indicated exposure conditions. Scale bars = 100 μm. (D) Quantification of MDC fluorescence intensity. LC3B abundance and MDC fluorescence represent static autophagy-associated measurements. (E) Representative RT-PCR agarose gel electrophoresis showing amplification products corresponding to four alveolar type II epithelial cell-associated marker genes in the isolated chicken embryonic lung epithelial cell preparations: LAMP3 (200 bp), SFTPC (164 bp), SFTPA1 (177 bp), and FABP3 (172 bp). M, 100-bp DNA ladder. The marker-gene analysis provides qualitative transcript-level evidence of the cellular phenotype and was not used to quantify gene expression or cell purity. For panels A-D, data are presented as mean ± SD from 3 independent cell-isolation experiments. Technical replicate wells within each independent isolation were averaged before statistical analysis, and each independent isolation was considered one biological replicate (n = 3). Statistical comparisons were performed using one-way ANOVA followed by Tukey’s multiple comparison test. Brackets indicate the pairwise comparisons shown in the figure. *P < 0.05, **P < 0.01, and ***P < 0.001.

MDC fluorescence was higher in the sequential group than in the control and single-exposure groups (Figure 3C and D), indicating greater accumulation of MDC-positive, autophagy-associated structures. Together with the higher LC3B signal (Figure 3A and B), this result shows accumulation of autophagy-related markers/structures under the sequential schedule. Because autophagic flux was not measured, the data cannot distinguish increased autophagosome formation from impaired downstream degradation.

To further characterize the phenotype of the isolated chicken embryonic lung epithelial cells, conventional RT-PCR and agarose gel electrophoresis were performed to examine four alveolar type II epithelial cell-associated marker genes. Amplification products consistent with the expected sizes of LAMP3 (200 bp), SFTPC (164 bp), SFTPA1 (177 bp), and FABP3 (172 bp) were detected in the isolated cell preparations (Figure 3E). Together with the alkaline phosphatase BCIP/NBT staining results, these findings provide additional transcript-level evidence supporting a type II-associated epithelial phenotype.

### NAC and CBIQ partially attenuate selected responses associated with sequential PM2.5 and APEC exposure

To examine whether the observed responses were pharmacologically modifiable, cells were pretreated with NAC or CBIQ before sequential PM2.5-followed-by-APEC exposure. Both interventions partially attenuated selected inflammatory and redox-related responses relative to sequential exposure alone (Figure 4).

**Figure 4:**
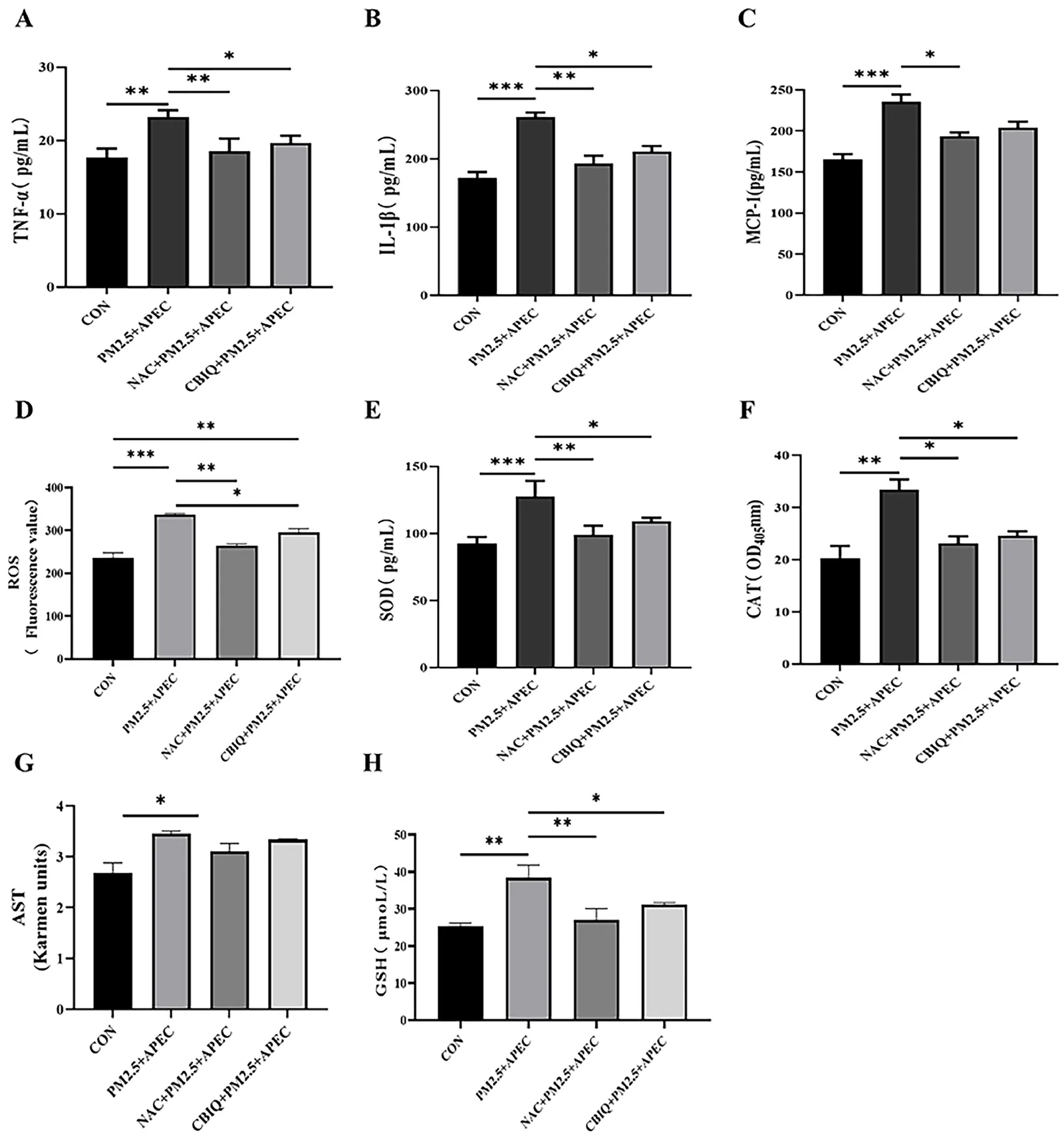
NAC and CBIQ partially modify selected inflammatory, redox-related, and injury-associated responses under sequential PM2.5-followed-by-APEC exposure in chicken embryonic type II-like lung epithelial cells. Cells were pretreated with NAC or CBIQ before sequential PM2.5 followed by APEC exposure. (A–C) Concentrations of TNF-α, IL-1β, and MCP-1 in cell-culture supernatants. (D) ROS-associated fluorescence signal. (E–H) SOD, CAT, AST, and GSH, respectively. Data are presented as mean ± SD from 3 independent cell-isolation experiments. Technical replicate wells within each independent isolation were averaged before statistical analysis, and each independent isolation was considered one biological replicate (n = 3). Statistical comparisons were performed using one-way ANOVA followed by Tukey’s multiple comparison test. Brackets indicate the pairwise comparisons shown in the figure. *P < 0.05, **P < 0.01, and ***P < 0.001. Abbreviations: APEC, avian pathogenic *Escherichia coli*; AST, aspartate aminotransferase; CAT, catalase; CBIQ, 4-chlorobenzo[f]isoquinoline; GSH, glutathione; MCP-1, monocyte chemoattractant protein 1; NAC, N-acetyl-L-cysteine; ROS, reactive oxygen species-associated fluorescence signal; SOD, superoxide dismutase; TNF-α, tumor necrosis factor α.

Pretreatment with either NAC or CBIQ lowered TNF-α, IL-1β, and MCP-1 concentrations relative to sequential exposure alone, although the magnitude of the response differed among mediators (Figure 4A–C). The ROS-associated fluorescence signal was also lower after NAC or CBIQ pretreatment than after sequential exposure alone (Figure 4D). SOD and CAT activities and GSH levels were modified by the interventions, with selected measurements shifting toward control values (Figure 4E, F, and H). AST, assessed as a cell injury-associated indicator, was lower after either intervention than after sequential exposure alone (Figure 4G).

Overall, NAC and CBIQ partially attenuated selected inflammatory, redox-related, and injury-associated measurements observed under the sequential schedule. These interventions demonstrate pharmacological modifiability of selected endpoints, but they do not identify a unique upstream mechanism because NAC and CBIQ can affect multiple cellular processes.

### NAC and CBIQ partially attenuate selected molecular responses associated with sequential PM2.5 followed by APEC exposure

To determine whether the molecular changes observed under the sequential-exposure schedule were pharmacologically modifiable, cells were pretreated with NAC or CBIQ before PM2.5 exposure followed by APEC challenge. Figure 5 summarizes transcript abundance of CFTR, PI3K, AKT, mTOR, and NF-κB p65, together with total-protein abundance of CFTR, PI3K, AKT, mTOR, LC3B, IκBα, and NF-κB p65.

**Figure 5:**
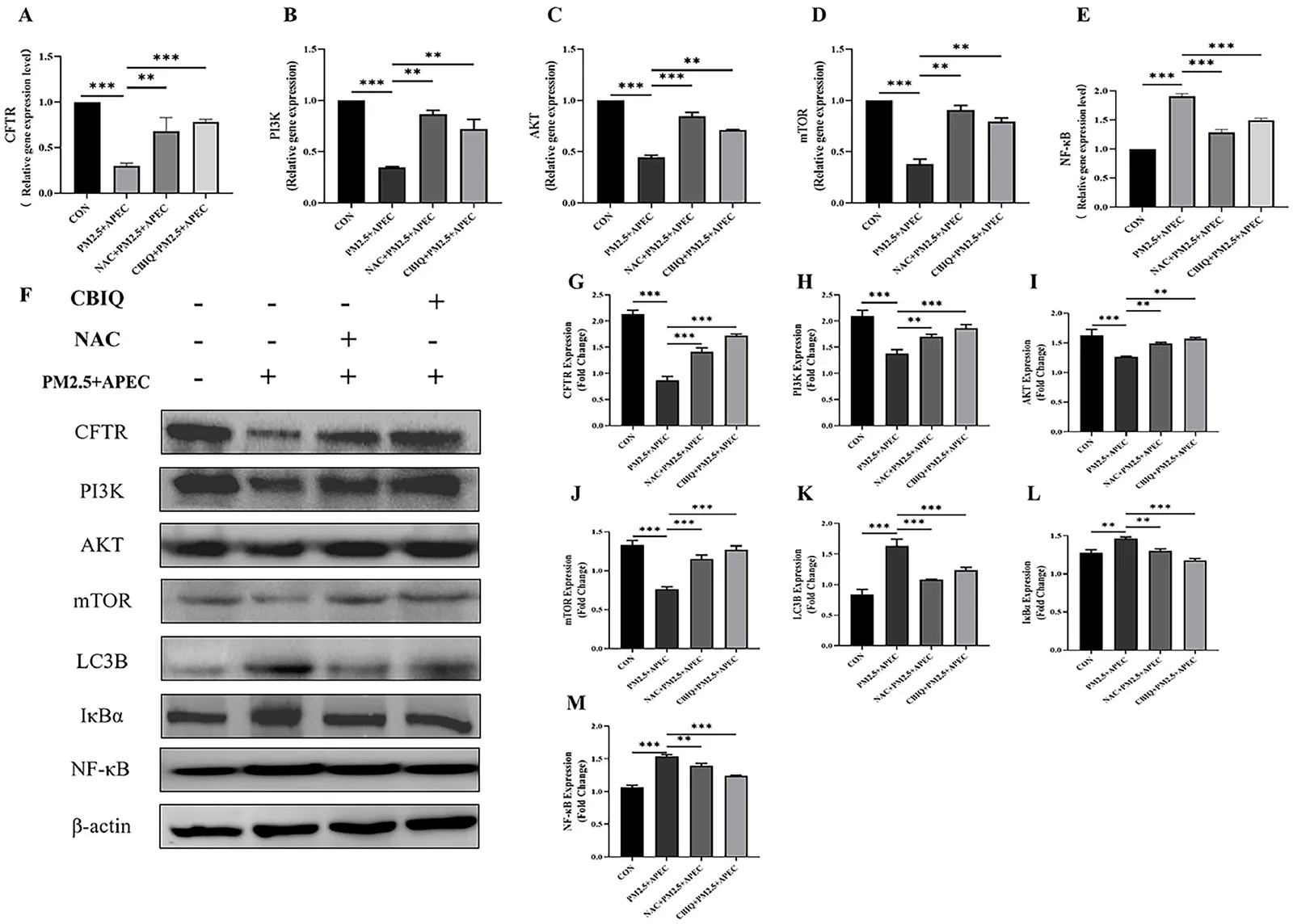
NAC and CBIQ partially modify transcript and total-protein abundance under sequential PM2.5-followed-by-APEC exposure in chicken embryonic type II-like lung epithelial cells. Cells were pretreated with NAC or CBIQ before sequential PM2.5 followed by APEC exposure. (A–E) Relative transcript abundance of CFTR, PI3K, AKT, mTOR, and NF-κB p65. (F) Representative Western blot images of CFTR, PI3K, AKT, mTOR, LC3B, IκBα, NF-κB p65, and β-actin. (G–M) Densitometric quantification of CFTR, PI3K, AKT, mTOR, IκBα, LC3B, and NF-κB p65, respectively, normalized to β-actin. Data are presented as mean ± SD from independent cell-isolation experiments (n = 3). Technical replicate wells within each independent isolation were averaged before statistical analysis, and each independent isolation was considered one biological replicate. Statistical comparisons were performed using one-way ANOVA followed by Tukey’s multiple comparison test. Brackets indicate the pairwise comparisons shown in the figure. *P < 0.05, **P < 0.01, and ***P < 0.001.

Sequential exposure was associated with lower CFTR, PI3K, AKT, and mTOR transcript abundance than the control treatment, whereas NAC or CBIQ pretreatment shifted these measures upward to varying degrees (Figure 5A–D). A similar pattern was observed at the protein level: total CFTR, PI3K, AKT, and mTOR abundance was lower after sequential exposure and was increased to varying extents by NAC or CBIQ pretreatment in the comparisons shown in Figure 5F–J. Thus, both interventions partially attenuated selected CFTR- and PI3K/AKT/mTOR-related expression changes associated with sequential exposure.

In contrast, NF-κB p65 transcript and total-protein abundance were higher under the sequential-exposure schedule, and both interventions attenuated selected increases (Figure 5E, F, and M). LC3B and IκBα total-protein abundance also differed among treatments, with higher values under sequential exposure and lower values after NAC or CBIQ pretreatment (Figure 5F, K, and L). The magnitude of these changes varied according to the measured endpoint and pharmacological intervention.

Overall, NAC and CBIQ shifted selected transcript and total-protein measurements toward control-associated values under the sequential schedule. This pharmacological modifiability is compatible with redox- and CFTR-related contributions but does not establish a unique upstream mechanism.

### Sequential PM2.5 followed by APEC is associated with qualitative lung histology and serum inflammatory/redox responses in chicks

Representative H&E-stained sections from control chicks showed comparatively preserved pulmonary architecture, whereas PM2.5-derived material or APEC alone was accompanied by variable histological changes. Representative sections from the sequential group showed more extensive qualitative abnormalities, including disruption of pulmonary tissue organization and inflammatory-appearing features (Figure 6A–P). No deaths occurred in any group during the 14-d exposure period, and routine daily monitoring did not identify overt severe clinical illness. A prespecified numerical clinical score was not used. Histology was descriptive; because a standardized per-bird lesion-scoring protocol was not part of the original study, no quantitative lesion-severity score is reported.

**Figure 6:**
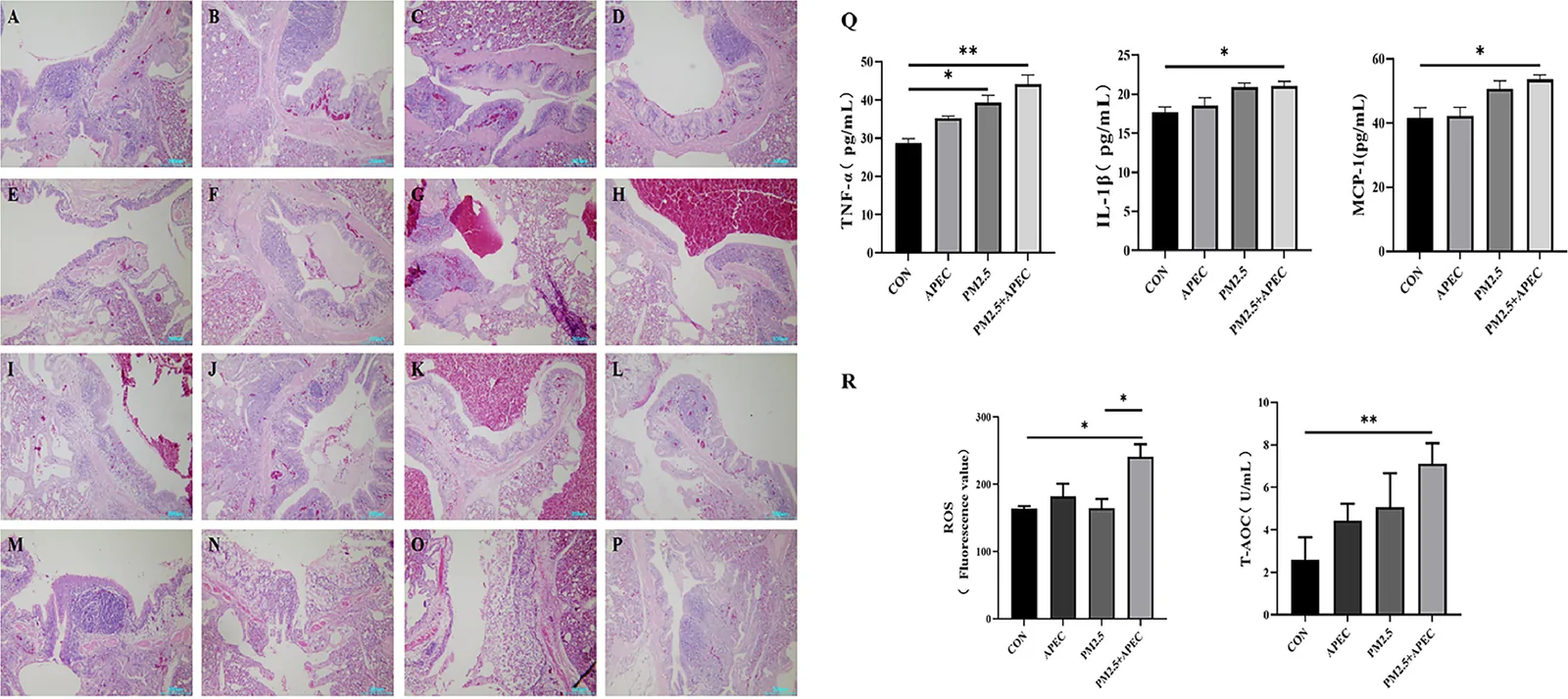
Qualitative lung histology and serum inflammatory/redox-related responses under the chick exposure schedules. (A–P) Representative H&E-stained lung sections from control, PM2.5, APEC, and sequential PM2.5-followed-by-APEC groups. Images were acquired at 100 × magnification, corresponding to scale bars of 200 μm. Histology was evaluated descriptively; no standardized per-bird lesion-severity score is reported. (Q) Serum TNF-α, IL-1β, and MCP-1 concentrations. (R) Serum ROS-associated fluorescence signal (left panel; fluorescence value) and total antioxidant capacity (T-AOC; right panel; U/mL). Chicks arrived at 7 d of age, treatment began at 10 d of age, and samples were collected after the 14-d exposure period. Values are mean ± SD (n = 10 chicks/group). Statistical notation is defined in the figure panels. Abbreviations: APEC, avian pathogenic *Escherichia coli*; MCP-1, monocyte chemoattractant protein 1; ROS, reactive oxygen species-associated fluorescence signal; T-AOC, total antioxidant capacity; TNF-α, tumor necrosis factor α.

Serum inflammatory and redox-related indices were examined as complementary in vivo endpoints. Serum TNF-α, IL-1β, and MCP-1 concentrations were higher in the sequential-exposure group than in the control group, with additional differences from the single-exposure groups detected for selected mediators (Figure 6Q). The serum ROS-associated fluorescence signal and T-AOC values were higher in the sequential-exposure group than in the control group (Figure 6R).

Overall, the serum inflammatory/redox patterns and qualitative pulmonary histology were directionally consistent with selected cellular findings. These observations provide complementary in vivo context but do not quantify clinical disease or lesion severity.

### Sequential PM2.5 followed by APEC is associated with altered CFTR- and inflammation-related molecular abundance in chick lung

To assess whether molecular patterns observed *in vitro* were also present *in vivo*, transcript and total-protein abundance were measured in lung tissue from chicks assigned to the four schedules. Relative to the control and, for selected endpoints, single-exposure groups, the sequential group showed differences in CFTR, PI3K, AKT, mTOR, NF-κB-related measurements, and LC3B (Figure 7).

**Figure 7:**
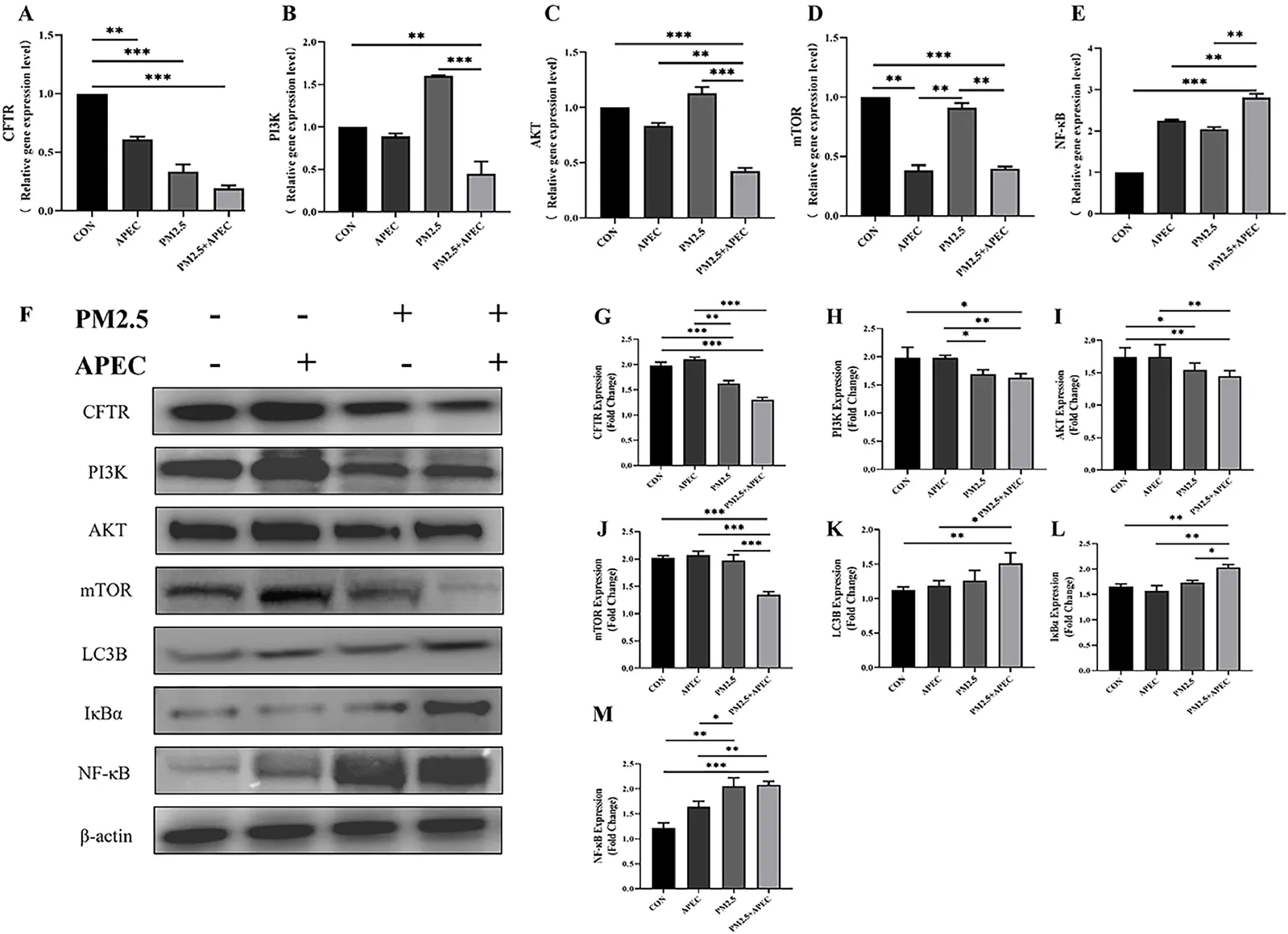
CFTR-related, inflammation-related, and autophagy-associated transcript/total-protein abundance in chick lung under the tested exposure schedules. (A–E) Relative transcript abundance of CFTR, PI3K, AKT, mTOR, and NF-κB p65. (F) Representative Western blots. (G–M) Densitometric quantification of total CFTR, PI3K, AKT, mTOR, LC3B, IκBα, and NF-κB p65 abundance normalized to β-actin. Chicks arrived at 7 d of age and treatment began at 10 d of age. Values are mean ± SD (n = 10 chicks/group). Statistical notation is defined in the figure panels. Abbreviations: APEC, avian pathogenic *Escherichia coli*; CFTR, cystic fibrosis transmembrane conductance regulator; LC3B, microtubule-associated protein 1 light chain 3 beta; NF-κB p65, nuclear factor κB subunit p65; PI3K, phosphoinositide 3-kinase; AKT, protein kinase B; mTOR, mechanistic target of rapamycin.

At the transcript level, CFTR, PI3K, AKT, mTOR, and NF-κB p65 abundance differed among treatment schedules (Figure 7A–E), with the significant pairwise comparisons shown in the individual panels. Total-protein abundance of CFTR, PI3K, AKT, mTOR, LC3B, IκBα, and NF-κB p65 also differed among schedules (Figure 7F–M). Selected lung molecular patterns were directionally consistent with the cell model, providing complementary evidence that the tested sequential schedule was associated with a reproducible response pattern across the two experimental systems.

## Discussion

Under the prespecified schedules used here, PM2.5-derived material followed by APEC was associated with the largest changes for several inflammatory and redox-related endpoints in chicken embryonic type II-like lung epithelial cells and with selected serum and lung changes in chicks. The same schedule was accompanied by lower CFTR abundance, altered abundance of PI3K, AKT, mTOR, IκBα, and NF-κB p65, and greater LC3B/MDC-associated signals. NAC and CBIQ shifted selected cellular endpoints toward control-associated values. These observations define a response pattern under the tested schedules; they do not, by themselves, establish a PM2.5 × APEC interaction, particle-specific toxicity, or a complete causal pathway.

Poultry-house PM2.5 is compositionally complex. Studies of broiler-house PM2.5 identify organic and inorganic constituents, diverse microorganisms, and measurable endotoxin (Dai et al., 2020), and recent Poultry Science work confirms substantial bacterial and fungal diversity in poultry-house fine-particle aerosols (Wang et al., 2024). In the present study, material collected in the PM2.5 sampling fraction was eluted, lyophilized, pooled, and autoclaved before administration. We did not measure post-processing particle-size distribution, elemental/ionic composition, microbiological composition, or endotoxin. Because autoclaving is not a depyrogenation procedure, heat-stable lipopolysaccharide may have remained. The inflammatory and redox-related responses therefore cannot be attributed specifically to the particulate fraction and may reflect particle-derived, endotoxin-related, or other heat-stable microbial components. This limitation is central to interpretation and is why the material is described as processed PM2.5-derived material rather than as a fully characterized PM2.5 particle preparation (Table 2).

**Table 2:** Treatment groups and exposure protocols for the cell and chick experiments.

**Model**	**Group**	**Treatment**	**Details**
Cell	Control	Corresponding vehicle/medium	Harvest at 4.5 h
Cell	PM2.5	Processed PM2.5-derived material, 0.50 mg/mL	Agent present over the 4.5-h experimental interval; harvest at 4.5 h
Cell	APEC	MOI 100:1	Agent present over the 4.5-h experimental interval; harvest at 4.5 h
Cell	Sequential PM2.5-APEC exposure	PM2.5-derived material for 2 h, then APEC for 2.5 h	Harvest at 4.5 h; agent-specific exposure duration is not matched to single-agent schedules
Chick	Control	PBS, 200 μL/chick, d 1–14	Dropwise at nares (100 μL/nostril); treatment begins at 10 d of age
Chick	PM2.5	1 mg/mL, 200 μL/chick (0.20 mg/administration), d 1–14	Dropwise at nares; daily routine clinical monitoring
Chick	APEC	1 × 10⁸ CFU/mL, 200 μL/chick (2 × 10⁷ CFU/administration), d 1–14	Dropwise at nares; daily routine clinical monitoring
Chick	Sequential PM2.5-APEC exposure	PM2.5-derived material, d 1–7; APEC, d 8–14 (same concentration/volume as single groups)	Sequential dropwise exposure; schedules are nonparallel and not factorial; samples collected after d 14
Cell (intervention)	Sequential exposure + NAC	5 mM NAC for 1 h before PM2.5; then 2 h PM2.5-derived material + 2.5 h APEC	Harvest at 4.5 h after exposure start
Cell (intervention)	Sequential exposure + CBIQ	10 μM CBIQ for 1 h before PM2.5; then 2 h PM2.5-derived material + 2.5 h APEC	Final DMSO <0.1%; harvest at 4.5 h after exposure start

The second major limitation is the exposure design. In chicks, the PM2.5-only and APEC-only groups received their respective agent on d 1 to 14, whereas the sequential group received PM2.5-derived material on d 1 to 7 and APEC on d 8 to 14. In cells, all groups shared a 4.5-h harvest time, but the sequential group received PM2.5-derived material for 2 h followed by APEC for 2.5 h, whereas single-agent cultures were exposed over the 4.5-h interval. Thus, exposure type, duration, timing, and cumulative agent-specific exposure are not fully matched. Larger responses in the sequential group cannot be interpreted as statistical synergy, causal priming, or evidence that prior PM2.5 exposure specifically potentiated APEC. A matched factorial design with harmonized exposure windows is required to estimate an interaction directly.

The concurrent increases in proinflammatory mediators and the ROS-associated signal indicate that inflammatory and redox-related responses occurred in parallel. Higher SOD, CAT, GSH, and serum T-AOC assay values should not be interpreted as direct evidence of reduced oxidative burden. These changes may reflect associated or compensatory redox-related responses; however, the T-AOC measurement alone does not establish an improvement in functional antioxidant defense. PM2.5-associated oxidative injury and autophagy-related responses have been reported in epithelial and macrophage systems (Su et al., 2017; Zhu et al., 2018; Han and Zhuang, 2021), and a recent broiler study linked inhaled PM2.5 with lung inflammatory responses and systemic/gut changes (Zhou et al., 2024). NAC attenuated selected endpoints in our cell model, which is compatible with a redox-sensitive component of the response; however, NAC has multiple biochemical actions and the present experiments do not establish ROS as the sole upstream driver.

CFTR contributes to epithelial ion transport, airway-surface-liquid homeostasis, and inflammatory regulation (Saint-Criq and Gray, 2017), and environmental pollutants can alter CFTR expression or function in respiratory epithelia (Nguyen et al., 2021; Stermann et al., 2022; Liu et al., 2024). Lower CFTR abundance under the sequential schedule, together with partial attenuation of selected endpoints by CBIQ, is compatible with CFTR-related processes. However, CFTR channel activity, chloride transport, membrane localization, and airway-surface-liquid physiology were not measured, and CBIQ is not a completely selective CFTR agonist (Murthy et al., 2005). The findings therefore do not establish CFTR as the direct mediator of the inflammatory or redox phenotype.

Likewise, changes in total PI3K, AKT, mTOR, IκBα, and NF-κB p65 abundance do not demonstrate activation or inhibition of PI3K/AKT/mTOR or NF-κB signaling. Such activity-dependent interpretation would require phosphorylation-state measurements, IκBα turnover, and/or NF-κB p65 nuclear translocation. Higher LC3B abundance and MDC fluorescence indicate accumulation of autophagy-associated markers/structures but cannot distinguish increased autophagosome formation from impaired clearance because autophagic flux was not measured. The present molecular data are therefore best viewed as expression/abundance profiles that identify candidate processes for subsequent functional testing rather than as proof of pathway activation.

The chick experiment provides *in vivo* context but not a complete disease-severity assessment. APEC disease expression is influenced by host status and environmental stressors (Alber et al., 2021; Nawaz et al., 2024). In our study, no deaths occurred and routine daily monitoring did not identify overt severe clinical illness, but a prespecified numerical clinical score was not used. Body weight/growth performance, gross or airsac lesion scores, tissue APEC burden, bacteremia, and standardized per-bird histologic lesion scores were not collected. The H&E material was therefore retained as qualitative histology rather than converted retrospectively into a numerical severity score from representative archived fields. These missing phenotypic and microbiological endpoints prevent us from determining whether the molecular and serum changes corresponded to clinically meaningful colibacillosis severity or production loss.

Additional limitations include the absence of a dedicated cell-viability/LDH assay, the use of AST only as a nonspecific injury-associated readout, and incomplete protein-level lineage validation of the embryonic type II-like lung epithelial cells. The biological replicate number for cell-isolation experiments was three independent isolation batches, with technical wells averaged within each batch. Taken together with the uncharacterized processed PM2.5-derived material and nonmatched exposure schedules, these constraints narrow the conclusions to descriptive associations under the conditions tested. Future work should prioritize endotoxin and physicochemical characterization, a harmonized factorial exposure design, direct CFTR/pathway/autophagic-flux assays, stronger epithelial lineage validation, and prespecified clinical, lesion, growth, and bacterial-load endpoints.

The phenotypic characterization of the isolated chicken embryonic lung epithelial cells was strengthened by combining alkaline phosphatase BCIP/NBT staining with RT-PCR detection of LAMP3, SFTPC, SFTPA1, and FABP3 transcripts. These markers are consistent with the molecular features of chicken alveolar type II epithelial populations described in recent single-cell transcriptomic studies (Gerner-Mauro et al., 2025). Nevertheless, conventional RT-PCR of bulk cell preparations cannot determine whether all four markers are co-expressed within individual cells or establish the purity of the isolated population. In the absence of protein-level immunophenotyping or flow cytometric characterization, the designation "type II-like lung epithelial cells" remains appropriate.

In conclusion, sequential exposure to processed poultry-house PM2.5-derived material and APEC was associated with a pronounced inflammatory/redox response pattern and altered CFTR-related, inflammation-related, and autophagy-associated molecular measurements in chicken cell and chick models under the schedules tested. Pharmacological interventions modified selected cellular endpoints, but the study does not establish particle-specific effects, a PM2.5 × APEC interaction, causal priming, or pathway activation. The findings support further study of how poultry-house bioaerosol exposures and APEC pressure intersect, using fully characterized particulate preparations, matched factorial designs, and clinically relevant poultry-health endpoints.

## Funding

This work was supported by the Key Research and Development Plan of Shandong Province (2025CXGC010803); the Shandong Provincial Action Plan for Boosting Scientific and Technological Innovation in Rural Revitalization (2025TZXD017); the Young Scientists Fund of the Shandong Provincial Natural Science Foundation (ZR2024QC103, ZR2021QC167, and ZR2023MC049); the Young Scientists Fund of the National Natural Science Foundation of China (32202024); the Shandong Province Poultry Industry Technology System (SDAIT-11-10); and the Shandong Province Science and Technology-Based Small and Medium-Sized Enterprises Innovation Ability Improvement Project (2024TSGC0558). The funding agencies had no role in study design, data collection, data analysis or interpretation, manuscript preparation, or the decision to submit the work for publication.

## Author contributions

**Luna Li** contributed to the conception and design of the study, developed experimental procedures, conducted experiments, analyzed and organized the study data, prepared figures, and drafted the manuscript. **Cheng Bao** contributed to the conception and design of the study, developed experimental procedures, conducted and verified experimental work, analyzed the study data, prepared figures, and drafted the manuscript. **Chong Liu** contributed to the development of experimental procedures, conducted experiments, and collected and organized the study data. **Linlin Jiang** conducted experiments and participated in checking the reliability of the experimental work and study data. **Jiao Wang** conducted experiments and collected experimental data. **Xin Yu** contributed to the development of experimental procedures, conducted experiments, and collected experimental data. **Xinye Wang** conducted experiments, collected experimental data, and prepared figures to present the study findings. **Dechen Lu** conducted experiments and collected experimental data. **Guozhong Chen** contributed to the development of experimental procedures and provided essential resources for the study. **Hongwei Zhu** provided essential research resources and participated in verifying the experimental work and study data. **Haoxiang Li** analyzed and interpreted the study data and prepared figures to illustrate the findings. **Ziru Sun** collected and organized the study data and contributed to the coordination and management of the research project. **Xingxiao Zhang** contributed to the conception and design of the study, supervised and coordinated the research project, secured research funding, and critically reviewed and revised the manuscript for important intellectual content. **Jianlong Zhang** contributed to the conception and design of the study, supervised and coordinated the research project, secured research funding, and critically reviewed and revised the manuscript for important intellectual content. All authors critically reviewed and revised the manuscript for important intellectual content, reviewed and explicitly approved the final version for publication, and agree to take personal and public responsibility for all aspects of the work. All authors also agree to ensure that any questions concerning the accuracy or integrity of any part of the research are appropriately investigated and resolved.

## Data availability

The data that support the findings of this study are available from the corresponding author upon reasonable request.

## Declaration of AI and AI-Assisted Technologies in the Writing Process

During the preparation of this revised manuscript, the authors used ChatGPT (OpenAI) to assist with language editing, readability, and organization. After using this tool, the authors reviewed and edited the content as needed and verified the scientific content, data interpretation, and references. The authors take full responsibility for the content of the publication.

## Disclosures

The authors declare no competing financial or personal interests.
